# Myopia Is an Ischemic Eye Condition: A Review from the Perspective of Choroidal Blood Flow

**DOI:** 10.3390/jcm13102777

**Published:** 2024-05-09

**Authors:** Jiaul Baksh, Deokho Lee, Kiwako Mori, Yan Zhang, Hidemasa Torii, Heonuk Jeong, Jing Hou, Kazuno Negishi, Kazuo Tsubota, Toshihide Kurihara

**Affiliations:** 1 Department of Ophthalmology, Keio University School of Medicine, 35 Shinanomachi, Shinjuku-ku, Tokyo 160-8582, Japan; 2 Laboratory of Photobiology, Keio University School of Medicine, 35 Shinanomachi, Shinjuku-ku, Tokyo 160-8582, Japan; 3 Tsubota Laboratory, Inc., 34 Shinanomachi, Shinjuku-ku, Tokyo 160-0016, Japan

**Keywords:** choroidal blood flow, choroidal thickness, scleral hypoxia, light exposure, myopia

## Abstract

Myopia is a common refractive error that affects a large proportion of the population. Recent studies have revealed that alterations in choroidal thickness (ChT) and choroidal blood flow (ChBF) play important roles in the progression of myopia. Reduced ChBF could affect scleral cellular matrix remodeling, which leads to axial elongation and further myopia progression. As ChT and ChBF could be used as potential biomarkers for the progression of myopia, several recent myopia treatments have targeted alterations in ChT and ChBF. Our review provides a comprehensive overview of the recent literature review on the relationship between ChBF and myopia. We also highlight the importance of ChT and ChBF in the progression of myopia and the potential of ChT as an important biomarker for myopia progression. This summary has significant implications for the development of novel strategies for preventing and treating myopia.

## 1. Introduction

Myopia, or shortsightedness, is a condition in which visual images are focused in front of the retinal plane while accommodation is relaxed, which results in blurred distance vision. Myopia is a leading cause of refractive error, and it has recently become more prevalent worldwide. By 2050, approximately half of the world’s population is estimated to be myopic [1]. This estimation is different in East Asian countries, as the prevalence has already been reported to be close to 80% in the teenage group [2,3,4,5]. The economic burden due to this issue is also high; lost productivity due to vision impairment may have cost the global economy USD 244 billion in 2016 [6].

Myopia is traditionally treated with single-vision (SVn) glasses or contact lenses (CL). SVn glasses or CL improve visual performance rather than preventing or reversing axial elongation. Hence, the traditional treatments are not effective; they lead to frequent increases in refractive error and further progression to high myopia (HM). HM increases the risk of vision-threatening diseases, such as myopic macular degeneration (MMD), cataracts, retinal detachment, and glaucoma [7]. In Japan, MMD is estimated to cause vision impairment in approximately 200,000 people [8]. Therefore, understanding the precise mechanism of axial elongation in myopia is highly required. Studies have provided compelling evidence that multiple factors may be associated with the development of myopia: genetic and environmental factors, including near work and outdoor activities. Recent findings indicated that the choroid, specifically alterations in blood flow, may play an important role in axial elongation in animal models [9,10]. In human studies, myopic eyes have reported decreased choroidal vascularity and choriocapillaris blood flow [11,12].

**Figure 1:**
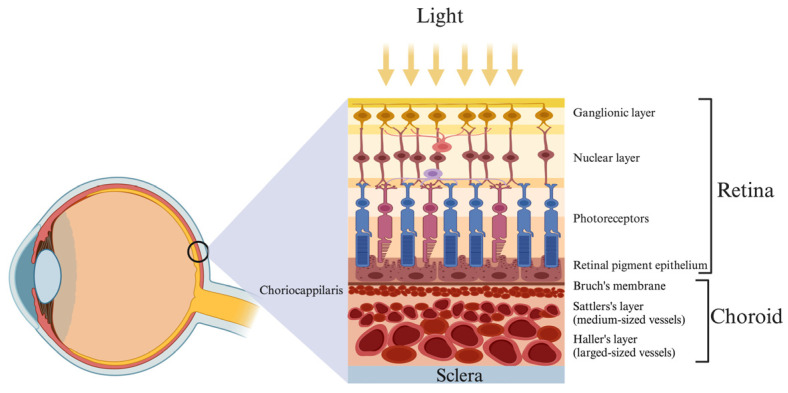
An illustration showcasing the intricate vascular network of the choroid, comprised of the choriocapillaris and medium- and large-sized blood vessels, in a healthy eye choroid. Created with graphics from ©BioRender (biorender.com).

The choroid is a highly vascularized structure posterior to the uvea, regulating approximately 85% of blood flow in the outer retina [13]. The primary source of nourishment for the outer retina is the choriocapillaris, a dense network of capillary beds of tiny blood vessels in the choroid. Interestingly, the choroidal circulation exhibits high blood flow, while the retinal circulation shows low blood flow. Notably, the regulation of blood flow differs between the two: retinal blood flow is mainly governed by local vasogenic factors, while choroidal blood flow is primarily controlled by sympathetic innervation. The choroid has five layers and is divided from the retinal pigment epithelium (RPE) by a thin membrane called Bruch’s membrane. The layers after that are the vascular choriocapillaris, Sattler’s, and Haller’s layers (Figure 1). Finally, there is the suprachoroidal layer, which separates the choroid from the sclera [14].

Optical coherence tomography (OCT) has become an invaluable tool in the field of ophthalmology. OCT allows for non-invasive, high-resolution imaging of the retina, optic nerve, and other ocular structures, providing detailed cross-sectional images for clinicians to assess the thickness, integrity, and characteristics of different retinal layers. In this aspect, OCT has also significantly improved patient care and therapeutic outcomes. Visualization of the choroid was difficult until the development of enhanced depth imaging (EDI) and swept-source OCT [15]. EDI enabled deeper penetration and full-thickness imaging of the choroid, which greatly contributes to understanding choroidal diseases.

In the general population, ChT is relatively constant until the age of 50 years, and rapid thinning is observed after the fifth decade of life. The average ChT varies between 200 and 400 μm and among different ethnicities (Table 1). In myopic eyes, a reduction is noted by 13.62 μm with each diopter increase in myopia [16]. Similarly, several researchers have reported a reduction in ChBF as a sign of myopia progression [17,18]. A decrease in ChBF could be considered hypoxia. A group from Wenzhou University has shown an integrated theory of hypoxia for myopia, which started this new field of research [19].

Therefore, we aimed to provide a recent literature review of the possible role of choroidal hypoxia in the development of myopia.

## 2. Choroidal Blood Flow Changes in Myopia

The choroid is characterized by its high degree of vascularity [13]. It receives arterial blood supply from both the long and short posterior ciliary arteries. The long posterior ciliary arteries branch out at the oraciliaris retina and enter the choroid, close to the optic nerve. In contrast, the short posterior ciliary arteries penetrate the sclera around the optic nerve. The medium-sized and large blood vessels are in the Sattler and Haller layers and are surrounded by nonvascular smooth muscles, melanocytes, and connective tissue elements. Its blood flow is controlled by the central nervous system; parasympathetic innervation is a vasodilator, whereas sympathetic innervation is a vasoconstrictor. It is locally regulated by intrinsic choroidal neurons located in the suprachoroid [29]. Given its extensive blood supply, changes in ChT may be linked to variations in ChBF.

OCT angiography (OCT-A) is a non-invasive imaging technique that helps in the measurement of choroidal alterations [30]. Recent work executed OCT-A for understanding how changes in ChBF could contribute to the development and progression of various ocular diseases, including myopia [31].

Recently, preclinical studies have provided insights into the relationship between choroidal blood perfusion (ChBP) and ChT in myopia [9,10,32,33,34,35]. Zhang et al. investigated this relationship using OCT-A in myopia models using guinea pigs and demonstrated that ChT might be related to ChBP [9]. Moreover, this group found that ChT and ChBP might rapidly recover after removing the myopia-inducing form of deprivation (FDM) or negative lens stimulation. A similar phenomenon (recovery of ChT and ChBP) has been reported in the chicks eyes in the FDM-induced animal model [33,34,35].

Research on human myopic subjects has shed further light on the relationship between the ChBF and ChT. Wakabayashi et al. demonstrated that choroidal thinning and a significant delay in choroidal refilling were seen in subjects with high myopia [36]. Vascular loss associated with choroidal thinning, irregular vascular shape, or ChBF was possibly considered one of the reasons. Similarly, studies showed a reduction in retinal capillary microvasculature and an increase in the area of flow deficit in the choriocapillaris in the eyes with greater myopia [37,38]. However, not all studies have demonstrated a clear correlation between the ChT and ChBF in myopic eyes. In adult, healthy Japanese individuals, no significant link between sub-foveal choroidal thickness (SfCT) and ChBF was reported [39]. Another study looked into the choriocapillaris area between the myopic and control eyes and concluded there was no significant change between them [40]. Chinese children with anisomyopia greater than 1.50 diopter showed reductions in choroidal vascularity and choriocapillaris blood perfusion in the eye with greater anisomyopia in comparison with those in the fellow eye [11].

Furthermore, preschool children with anisometropic hyperopic amblyopia had a thicker choroid, a larger total choroidal area, a larger luminal area, and a larger stromal area in the eye than children with anisometropic hyperopic in the eye [41]. Several factors may have an impact on the ChT and ChBF in the myopic eye. A study looked into changes in choroidal vascular caliber and reported that diurnal variations are the principal cause behind changes in the ChT [42]. Kim and colleagues reported that ocular perfusion pressure, critical in determining ocular blood flow, may have a strong relationship with subfoveal choroidal thickness in eyes with myopia [43]. Interestingly, a recent study reported changes in ChBF (measured via laser speckle flowgraphy) with positive defocus (+2.5 D) after short-term visual exposure (a 30-min movie viewed at 2 m) in adults. Notably, the aforementioned study did not report any significant change in the retinal or optic nerve head (ONH) blood flow [44]. Similarly, optical treatment for myopia control based on myopic defocus, such as defocus incorporating multiple segments (DIMS) spectacle lenses and orthokeratology (OK) contact lenses, demonstrated increased ChT [45,46]. Both lens designs exhibited a rapid increase in ChT after one week of use, with OK wearers also showing an increased choroidal vascularity index.

Various studies have revealed an association between ChT and ChBF. The choroid is vulnerable to changes in blood flow that can result in thickness variations. Consequently, a reduction in blood flow might lead to choroidal hypoxia and thinning, which cause axial elongation. Nonetheless, the correlation between ChBF and ChT in myopia is complex and requires further investigation. Further research is essential to gain a better understanding of its underlying pathophysiology.

## 3. Choroidal Change with Near Work

The relationship between near-work and myopia has been debated. The high prevalence of myopia in East Asian cities is due to several environmental risk factors, including education, increased near work, and less outdoor time. Although the association of near work with myopia needs more investigation, it has been highly supported by a meta-analysis [47].

When it comes to a relationship between ChT and accommodation, in 1995, Wallman et al. observed choroidal structural reshaping in chickens exposed to accommodative stimuli [48]. They found that chickens could alter their refractive states by shifting their retinas forward or backward. The choroid thickens in response to myopic defocus (image focused in front of the retina) or thins in response to hyperopic defocus (image focused behind the retina). This term is also referred to as choroidal accommodation. Studies have reported similar phenomena in animals such as guinea pigs, marmosets, and humans [13,49]. Recently, a novel near-work myopia model in guinea pigs showed a decrease in ChBP and ChT [50].

Studies investigating the relationship between accommodation and ocular changes have shown elongation in axial length and thinning of the choroid with accommodation [51,52,53]. During accommodation, inward forces of the ciliary muscle on the equator of the globe produce mechanical stretching of the globe, leading to eye globe expansion. Reportedly, the choroid thins rapidly in response to hyperopic defocus induced by a negative-powered lens or prolonged accommodation, and vice versa [51]. The authors also noted that choroidal thinning and axial elongation were more common in myopic eyes than in emmetropic eyes. However, the ChT returned to normal when the myopic defocus was removed. A recent study on adults undergoing excimer laser surgery reported an average increase of 34 microns ChT [54]. The increase was seen immediately after the surgery and was assumed to be caused by a decrease in the tension of the ciliary muscle. As the clarity of the retinal image improved after the surgery, the tension in these muscles decreased, leading to an increase in ChT.

Based on current reports, we hypothesized that during near work activity, the posterior eyeball may expand and temporarily stretch through thinning of the choroid layer, which in turn moves the retina backward [51,52,53]. This stretching and thinning of the choroid may cause an alteration in ChBF. This persistent disruption in blood flow could contribute to a hypoxic state within the choroid, ultimately fostering axial elongation [19]. More investigations are required to fully unravel the intricate relationship among activities like near work and accommodation and the associated changes in the choroid before definitive conclusions can be made.

## 4. Modulation of Choroidal Blood Flow Using Pharmacological Interventions

Numerous pharmacological therapeutic approaches for the progression of myopia have been performed. Recently, targeting ChBF through pharmacological drug interventions has emerged as a potential strategy to suppress the progression of myopia [19]. This approach involves altering choroidal blood flow to prevent the elongation of the eye and further reduce the risk of myopia. Recent studies showed anti-hypoxic drugs and vasodilators could inhibit the development of myopia.

In guinea pigs with FDM and mice with lens-induced myopia (LIM), the expression of hypoxia-inducible factor-1α (HIF-1α), a marker of tissue hypoxia, was increased in myopic sclera. Moreover, treatment with anti-hypoxia medications (salidroside and formononetin) reported inhibition in experimental myopia progression [19]. In form-deprived guinea pig eyes, these anti-hypoxic drugs suppress the upregulation of HIF-1α expression while simultaneously downregulating the expression of COL1α1. In addition, the same group reported that the intraocular injection of vasodilator prazosin can increase ChBP and inhibit myopia progression in guinea pigs [55]. This effect is related to the ability of the substance to reduce scleral hypoxia. These findings suggest that anti-hypoxia drugs may hold promise as potential treatments for myopia.

Our group reported crocetin, a well-known vasodilator, as a potential anti-myopia supplement [56,57]. Crocetin is a bioactive carotenoid extracted from *Crocus sativus*. It has antioxidant and vasodilatory properties. The vasodilatory effects have been shown to enhance blood flow in various organs, including the lungs, brain, and eyes [58,59,60]. Previous reports demonstrated that crocetin is also effective in treating eye diseases such as glaucoma and age-related macular degeneration [61,62]. In a multicenter randomized, double-blind, placebo-controlled trial on sixty-nine schoolchildren aged 6–12 years to assess the preventive effect of crocetin against myopia [57]. The results showed a significant reduction in myopia progression, a decrease in axial length, and an increase in ChT after 24 weeks. In addition, crocetin administration also showed a significant suppressive effect on axial length in a mouse model of LIM [56]. One of the mechanisms of action of crocetin involves blocking protein kinase C activity in vascular smooth muscle cells, which results in the release of nitric oxide (NO) in vascular endothelial cells [63]. This leads to thickening of the choroid, which helps maintain an anti-hypoxic environment and acts as a suppressive mechanism for axial elongation. Interestingly, the regions with habitual crocetin consumption had a low prevalence of myopia [64,65,66]. Although there is no direct evidence of improvement in ChBF with crocetin, crocetin may alter ChT by increasing ChBF. However, further research is needed to investigate the effects of crocetin on ChBF.

Recently, *Ginko biloba* has emerged as a promising candidate for mitigating myopia, as evidenced by its ability to significantly elevate ChT and ChBP levels (irrespective of myopic induction) while also demonstrating axial inhibition in LIM mice [67]. The efficacy of *Ginkgo biloba* extracts (GBEs) in enhancing blood circulation is well established [68]. Moreover, the confluence of GBE’s characteristics in enhancing ChBP with its concurrent inhibition of myopia progression strengthens the case for an inherent link between myopia and ChBP.

In our recent publication, we reported that the topical administration of bunazosin hydrochloride (BH) in LIM mice inhibits myopia progression through an associated increase in ChBP [69]. Furthermore, BH suppressed choroidal and scleral thinning, suggesting that the increased ChBP contributed to the maintenance of thickness. Notably, in studies involving healthy human subjects, the topical application of BH has been reported to enhance blood velocity in the ONH, retina, and choroid, with no significant impact on blood pressure or heart rate [70]. This empirical evidence provides valuable insights into the new potential intervention strategy for myopia (Table 2).

Atropine, an anti-cholinergic drug, has been widely used as a therapy for myopia control, which not only targets the sclera and retina but also affects the choroid and RPE [72,73,74,75]. In children, a low dose of 0.3% atropine can result in a positive change in sub-foveal choroidal thickness, with a 21 µm increase after one week, 26 µm after three months, and 21 µm after six months [76]. While using 1% atropine, the greatest attainable choroidal thickness was reported to be 24 µm in one week of administration, and a similar dosage resulted in a 15 µm change in the healthy eyes. Recent studies have reported that 0.5% atropine can prevent choroidal thinning induced by hyperopic blur in myopic eyes [74]. While higher doses of atropine were found to significantly increase ChT, the use of 0.01% atropine dosage, which is currently more popular worldwide as an anti-myopia drug, yielded mixed results in increasing ChT [77,78]. Moreover, a recent study looked at changes in the vasculature profile (choriocapillaris flow) of the choroid with 0.01% atropine in humans and found no significant change [79]. Overall, while atropine has shown potential as a myopia control therapy, further research is needed to determine the correlation between ChBF and atropine.

## 5. Choroidal Changes with Light Exposure

Various studies have shown that spending time outdoors could effectively slow the progression of myopia. Jones et al. first reported this finding, which has since been reported by many researchers [80,81,82,83]. Recognizing the potential benefits of this approach, many countries have fostered outdoor recreational opportunities for students. In Taiwan, an initiative to promote more outdoor time during recess for students and a year of implementation found that students who spent at least 11 h per week outside at light intensities of 1000 lux or greater experienced significantly less myopic shift and axial elongation than the control group [84]. Studies conducted in Australia, the UK, and the USA have also suggested that spending time outdoors could inhibit the progression of myopia [80,85,86]. Notably, this preventive effect appears to be associated with the overall time spent outside rather than any specific sports participation or activity [86].

Preclinical experimental models have been frequently used to investigate the relationship between light and ocular growth. Researchers have conducted studies on various monochromatic light wavelengths in vivo to assess their impact on eye growth. Bright light has been reported to prevent the development of experimental myopia [87,88]. Interestingly, studies also found that light exposure could result in choroidal thickening [89,90].

In the realm of human studies, there is a correlation between light exposure and an increase in ChT. In a study involving healthy young adults, a 30-min daily exposure over one week to morning light therapy through commercially available light therapy glasses, emitting direct blue-green light with a peak wavelength of 500 nm and an illuminance of 506 lux, led to a dramatic (5-µm) increase in ChT [91]. Another study also found an increase in ChT after a 2 h exposure to 1000 lux through light-emitting glasses [92]. Contrary to previous findings, a recent study revealed that young individuals exhibited choroidal thinning after a 2 h exposure to high-intensity light outdoors (6000 to 50,000 lux) [93].

It is noteworthy that different chromatic light variations have various effects on the ChT. Interestingly, different chromatic light variations exert varied effects on ChT; a recent report from the same group found that exposure to broadband light, red light, and darkness resulted in a notable decrease in ChT, whereas exposure to blue light showed minimal to no effect [94]. In contrast, a recent study reported that blue light with a wavelength of 420 nm had the most significant effect on increasing ChT, even in the presence of a positive defocus [95].

Our group found that violet light (VL) at 360–400 nm is an important component of outdoor light for inhibiting myopia progression [96]. In modern society, there has been a general decline in outdoor activities where the VL is naturally present. Simultaneously, the prevalence of UV light overprotection has led to no VL indoors. A recent randomized controlled trial (RCT) has shown that short-term exposure to violet light could be effective in inhibiting the progression of myopia and leading to a significant increase in the ChT [97]. We reported that exposure to VL in the 360–400 nm range can upregulate the gene early growth response protein (EGR-1) expression and slow myopia progression [98].

Furthermore, we found that violet-sensitive retinal ganglion cells (RGCs) via neuropsin (OPN5) may help prevent myopia progression through the maintenance of ChT in LIM mice [71]. Additionally, our recent report highlights that exposure to violet light leads to elevated expression levels of both EGR-1 mRNA and protein in the mouse retina and retinal cells (661 W), revealing a complex interaction between OPN5 and EGR-1 [99]. These findings suggest that exposure to natural light, including VL or various monochromatic lights, positively affects ChT in humans. However, further research is needed to fully understand the relationship between light exposure and changes in ChT and ChBF.

**Figure 2:**
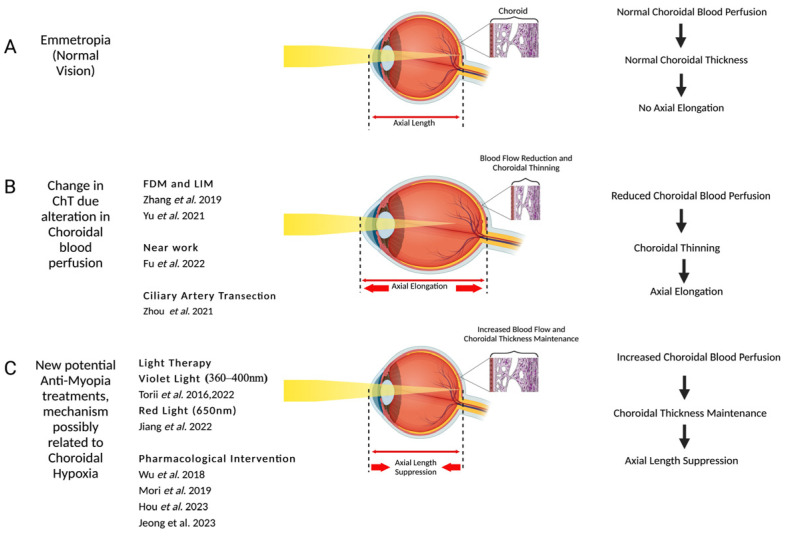
This figure shows the choroidal blood flow change and myopia correlation. (**A**) Normal choroidal perfusion and normal choroidal thickness; (**B**) studies proved a reduction in choroidal perfusion leads to a decrease in choroidal thickness and axial elongation; (**C**) anti-myopia treatment and increased perfusion lead to choroidal thickness maintenance and suppression of axial elongation. Created with graphics from ©BioRender (biorender.com) [9,10,19,32,50,56,57,67,69,97,98,100].

A recent RCT reported the effectiveness of repeated 650 nm, 1600 lux low-level red-light therapy (RLRL) for the treatment of myopia [100]. The results indicated RLRL may serve as a viable alternative treatment option for myopia control in children, which revealed a significant increase in ChT [101]. Importantly, RLRL exhibited good user acceptability and showed no evidence of causing any functional or structural damage. However, the group recently reported bilateral retinal damage in a 12-year-old patient, with a visual acuity drop of 2 lines for two weeks following five months of treatment [102]. Further studies are needed to investigate the RLRL mechanisms and modes of action underlying its impact on ChT and myopia progression (Figure 2).

Current research suggests that spending time outdoors, particularly under bright light conditions, can slow the progression of myopia and lead to an increase in ChT. Animal studies have shown that bright light prevents myopia and thickens the choroid, while human studies have observed a correlation between outdoor light exposure and increased ChT. Light plays a crucial role in the modulation of myopia, and research suggests that different wavelengths of short-term light exposure have the potential to slow down myopia progression and increase ChT. However, further investigation is required to fully comprehend the complex relationship between light exposure, ChT, and ChBF, as well as the underlying molecular mechanisms governing these processes. Advancing our understanding in these areas will pave the way for more targeted interventions and therapies aimed at effectively managing myopia. Continued research in this field holds promise for developing evidence-based strategies to mitigate the increasing prevalence of myopia and its associated complications.

## 6. Conclusions

In this current review article, we summarized changes in choroidal thickness related to variations in ChBF, the association of near work with myopia, and choroidal structural changes caused by accommodation. The choroid thickens in response to myopic defocus and thins with hyperopic defocus. Pharmacological interventions via anti-hypoxic drugs and vasodilators have been effective in inhibiting myopia development by altering ChBF. Crocetin, Ginko biloba, and Bunazosin could reduce myopia progression.

Moreover, by spending time outdoors, especially in bright light, myopia can be prevented. Human studies show a link between outdoor light exposure and increased ChT. VL is also effective in inhibiting myopia and increasing ChT. However, more research is needed to understand the relationship between light exposure, ChT, and ChBF, as well as the mechanisms behind VL effects on myopia and ChT.

The pilot study reported that VL is effective and safe. Currently, the final clinical research is underway, aiming to show the efficacy and safety of VL for the suppression of myopia progression. Upon successful validation, VL could serve as a practical alternative for individuals unable to spend time outdoors as a preventive measure against myopia.

Moreover, to enhance our comprehension of the pathogenesis of myopia, further research focusing on choroidal changes holds immense potential. Such investigations can contribute substantially to identifying novel therapeutic targets. We ensure our current summary can help understand the status of myopia and its underlying mechanisms and foster future studies to find promising strategies for myopia prevention.

## Figures and Tables

**Table 1:** Studies reported mean sub foveal choroidal thickness in different ethnicities.

Study Reference	Study Location	Number of Eyes	Age	Mean Axial Length	Subfoveal ChT
Read et al., 2013 [20]	Australia	104 eyes	10–15 years	22.98 ± 0.82 mm	337 ± 82 μm
Read et al., 2020 [21]		250 eyes	4–18 years	23.13 ± 0.73 mm	361 ± 74 µm
He et al., 2017 [22]	China	144 eyes	6–12 years	23.59 ± 1.09 mm	302 ± 63 μm
Xiong et al., 2017 [23]		3001 eyes	6–19 years	24.4 ± 1.31 mm	245 ± 66 μm
Bidaut-Garnier et al., 2014 [24]	France	348 eyes	3.5–14.9 years	22.3 ± 1.05 mm	341.96 ± 74.7 μm
Chabblani et al., 2015 [25]	India	255 eyes	5–18 years	23.55 ± 0.74 mm	312.1 ± 45.40 μm
Akhtar et al., 2018 [26]		30 eyes	12–18 years	-	327 ± 68 μm
Nagasawa et al., 2013 [27]	Japan	100 eyes	3–15 years	23.13 ± 1.37 mm	260 ± 57.2 μm
Ohsugi et al., 2018 [28]		64 eyes	3.6–5.8 years	21.90 ± 0.67 mm	301.8 ± 8.6 µm

**Table 2:** Influence of choroidal blood perfusion and choroidal thickness change in refraction in myopic animal models. (ChBP—Choroidal Blood Perfusion; ChT—Choroidal Thickness).

Study Reference	Species	Intervention	Change in ChBP	Change in ChT	Refractive Status
Zhang et al., 2019 [9]	Guinea Pigs	LIM (−4 D Lens) FDM	Decreased Decreased	Decreased Decreased	Myopic Shift
Zhou et al., 2021 [10]	Guinea Pigs	Temporal ciliary artery transection. Phenylephrine (Vasoconstrictor)	Decreased Decreased	Decreased Decreased	Myopic Shift
Yu et al., 2021 [32]	Guinea Pigs	LIM (−6 D Lens)	Decreased	Decreased	Myopic Shift
Fu et al., 2022 [50]	Guinea Pigs	Near work	Decreased	Decreased	Myopic Shift
Jiang et al., 2021 [71]	Mice	Violet Light	Not reported	Increased	Myopia Inhibition
Jeong et al., 2023 [69]	Mice	Bunazosin hydrochloride	Increased	Increased	Myopia Inhibition
Zhou et al., 2020 [55]	Guinea Pigs	Prazosin (Vasodilator)	Increased	Increased	Myopia Inhibition

## Data Availability

Not applicable.
